# Three-dimensional evaluation of facial attractiveness of skeletal Class II patient: comparative analysis after orthodontic and surgical simulations

**DOI:** 10.1590/2177-6709.30.5.e252584.oar

**Published:** 2026-01-09

**Authors:** Márcio Bastos de OLIVEIRA, Emanuel Braga RÊGO, Sara Ramos Braga SANTOS, Lucas Senhorinho ESTEVES, Maria Cristina Teixeira CANGUSSÚ, Jean Nunes dos SANTOS

**Affiliations:** 1 State University of Feira de Santana, Dentistry Course, Department of Pediatric Dentistry (Feira de Santana/BA, Brazil).; 2 Federal University of Bahia, Dentistry Course, Department of Orthodontics (Salvador/BA, Brazil).; 3 Federal University of Bahia, School of Dentistry, Department of Dentistry and Health (Salvador/BA, Brazil).

**Keywords:** Angle Class II malocclusion, Orthodontics, Orthognathic surgery, Má oclusão Classe II de Angle, Ortodontia, Cirurgia Ortognática

## Abstract

**Objective:**

The aim of this study was to assess the perception regarding the attractiveness of the face in patients with skeletal Class II and simulations of possible treatments for this condition.

**Methods:**

Dental scans, face scans and cone beam computed tomography (CBCT) of a male patient were used. These images were grouped in a software, where the original file was edited in order to achieve a profile that had the most pronounced skeletal and dental Class II (division 1) features, as well as the different proposed treatments: Compensatory orthodontics with extraction of the maxillary first premolars; Compensatory orthodontics associated with surgical advancement of the chin; and Orthodontics associated with combined orthognathic surgery of the maxilla, mandible and counterclockwise rotation of the occlusal plane. These simulations were presented in the form of videos and accompanied by a questionnaire regarding the attractiveness.

**Results:**

The videos were analyzed by 275 evaluators: 109 orthodontists, 91 oral and maxillofacial surgeons and 165 laypersons. The collected data were submitted to analysis of variance (ANOVA) and Student’s *t* tests, with a significance level of 95%. Individuals with higher levels of education were more rigorous in their aesthetic evaluation, but the most attractive face for all groups was achieved by orthodontic preparation associated with combined orthognathic surgery, while the least attractive was skeletal Class II without treatment.

**Conclusions:**

This study indicate that the face resulting from a skeletal Class II is aesthetically unfavorable and the treatments generate better aesthetic results, especially the orthodontic preparation associated with combined orthognathic surgery.

## RESUMO

**Objetivo:**

O objetivo deste estudo foi avaliar a percepção da atratividade facial em pacientes com Classe II esquelética e simular possíveis tratamentos para essa condição.

**Métodos:**

Foram utilizados escaneamentos dentários, escaneamentos faciais e tomografia computadorizada de feixe cônico (TCFC) de um paciente do sexo masculino. Essas imagens foram agrupadas em um software, onde o arquivo original foi editado para obter um perfil que apresentasse as características esqueléticas e dentárias de Classe II (1ª divisão) mais pronunciadas, bem como os diferentes tratamentos propostos: Ortodontia compensatória com extração dos primeiros pré-molares superiores; Ortodontia compensatória associada ao avanço cirúrgico do mento e Ortodontia associada à cirurgia ortognática combinada de maxila, mandíbula e rotação anti-horária do plano oclusal. Essas simulações foram apresentadas em forma de vídeos e acompanhadas de um questionário sobre atratividade.

**Resultados:**

Os vídeos foram analisados por 275 avaliadores: 109 ortodontistas, 91 cirurgiões bucomaxilofaciais e 165 leigos. Os dados coletados foram submetidos aos testes de Análise de Variância (ANOVA) e t de Student, com nível de significância de 95%. Indivíduos com maior escolaridade são mais rigorosos em sua avaliação estética, mas a face mais atraente para todos os grupos foi alcançada pelo preparo ortodôntico associado à cirurgia ortognática combinada, enquanto a menos atraente foi a Classe II esquelética sem tratamento.

**Conclusões:**

Este estudo indica que a face resultante de uma Classe II esquelética é esteticamente desfavorável e os tratamentos geram melhores resultados estéticos, especialmente o preparo ortodôntico associado à cirurgia ortognática combinada.

## INTRODUCTION

Stability and function are core aspects of orthodontic planning to ensure treatments that not only correct malocclusion, but also promote aesthetic gains and a better quality of life for the patient. However, growth patterns can lead to the development of malocclusions and aesthetically unbalanced faces. This negatively influences the individual’s self-perception and social interaction.^1^ ^-^ ^4^

Skeletal Class II malocclusion, especially in the first division, can compromise the perception of attractiveness. This category is characterized by protrusion of the upper front teeth and lips, thus resulting in an even more convex profile and impaired facial aesthetics. Therefore, the in-depth understanding of the aesthetic result of the therapeutic possibilities becomes even more relevant in these cases.^5^ ^-^ ^10^

Previous studies have investigated the facial variations resulting from skeletal Class II and the self-assessment of attractiveness based on the facial profile. Questionnaires and photographs with two-dimensional simulations in editing programs were used as assessment methods.^5^ ^,^ ^11^ ^,^ ^12^ However, two-dimensional methods for complete facial analysis are unfeasible because individuals usually interact with each other in a dynamic, three-dimensional (3D) way.^13^ ^-^ ^15^

The latest digital technologies, such as CBCT 3D facial scanning, and digital dental models, have enabled the face to be analyzed with greater detail and precision. These tools ensure that the outcome of a treatment can be studied before it is performed. However, these new resources have not yet been investigated for this type of assessment.^16^

The study aimed to assess the perception of orthodontists, oral and maxillofacial surgeons, and laypeople about the facial attractiveness of patients with skeletal Class II and the simulation of three-dimensional treatments for this condition. 

## MATERIAL AND METHODS

After approval by the Ethics Committee, a patient was selected from the database of a oral and maxillofacial surgeon. The following inclusion criteria were considered: 1) complete orthodontic records, including CBCT, digital models of the maxillary and mandibular arches, and facial scanning; 2) no evident asymmetries; 3) complete dental arches; 4) no prior surgeries or any facial aesthetic procedures; 5) skeletal Class II diagnosis, with an ANB angle greater than 6°, and Angle Class II, first division. The patient signed a consent form for the use of images and authorized the use of all or any part of the documentation to serve as an object of evaluation.

## ACQUISITION OF IMAGING TESTS

The following exams were used for this study: CBCT scans, digital models, and facial scans. The CBCT scans were performed using a Philips Brilliance 64 CBCT scanner CH (Koninklijke Philips N.V, Amsterdam, Netherlands), with 0.9-mm thick slices, 0.44mm spacing, 120Kv and 100 MAS. Digital models were acquired using a i-Teroelement 2 (Align Technology, Inc., San José, California, USA), using a tip measuring 338.5 mm in length, 552 mm in width and 625 mm in depth. The scanner has an output of 100-240 VAC - 50/60 Hz - 200VA and an operating temperature ranging from 16 to 26ºC. Finally, the facial scan was carried out by having the patient’s Frankfurt horizontal plane and bipupillary line parallel to the ground. A 7MP TrueDepth camera (Apple Inc., Cupertino, California, USA) was used, with the following settings: portrait lighting (beta version), 1080p HD video recording, retina flash, f/2.2 aperture, with wide color tone capture for photos and live photos, automatic HDR (High Dynamic Range), backlight sensor, face and body detection, automatic image stabilization, continuous mode and exposure control. The images were exported in .stl (for CBCT scans and digital models) and .obj (for facial scans) formats. 

## ACQUISITION OF VIDEOS

**Figure 1:**
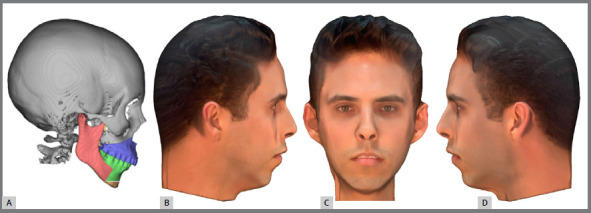
A) Image with tomography and digital models. B, C, D) Complete images with face.

The imaging exams obtained were grouped using Dolphin software (Dolphin Imaging & Management Solutions, Chatsworth, California) and manipulated to create four different clinical situations. The first was skeletal Class II, with a chin-neck length of 45mm and a nasolabial angle of 104,9°. This profile has the most pronounced Class II (first division) features and shows the largest interlabial step in the sagittal view (Fig. 1).^17^ ^-^ ^19^

**Figure 2:**
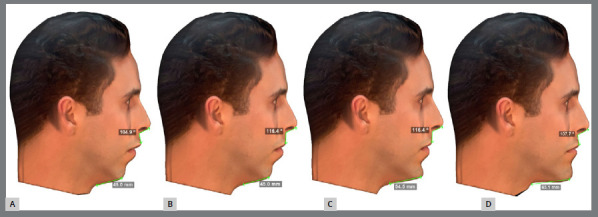
Profile image of the four faces generated by the simulations. **A)** Face with a Class II profile and its most pronounced features, without treatment. **B)** Face with an increased nasolabial angle (116.4º), simulating incisor retraction, resulting from a compensatory Class II orthodontic treatment. **C)** Face generated by an orthodontic-surgical treatment, of orthodontic compensation with retraction of the anterior teeth and surgical advancement of only the chin, generating a chin-neck length of 54.5 mm. **D)** Face representing a combined orthodontic-surgical treatment, with orthodontic alignment and leveling, associated with orthognathic surgery involving the maxilla, mandible, chin and rotation of the occlusal plane in the counterclockwise direction, generating a nasolabial angle of 107.7º and a chin-neck length of 63.1 mm.

Then, we conducted three skeletal Class II treatment simulations: Compensatory orthodontics with extraction of the maxillary first premolars; Compensatory orthodontics associated with surgical advancement of the chin; and Orthodontics associated with combined orthognathic surgery of the maxilla, mandible and counterclockwise rotation of the occlusal plane. In the end, four different clinical situations were made: the skeletal Class II (first division) without treatment and three possible treatments. All of them were exported and only showed the soft tissue coverage of the face, with no visible changes to bones or teeth. This process ensured a focus on the treatment outcome, regardless of the orthodontic or surgical resources required to achieve this goal (Fig. 2).

These simulations were saved as videos in .mp4 format, each lasting 10 seconds. The footage showed the patient’s head rotating from right to left, to allow a dynamic assessment of the face from all angles.

These videos were made available online, by email and phone calls, to orthodontists, oral and maxillofacial surgeons, and laypeople through the Google platform (www.docs.google.com). The orthodontists, oral and maxillofacial surgeons, and laypeople had their contacts randomly selected from the databases of dental councils in the state of Bahia (Brazil) and from the school clinics of dental education institutions, respectively. Participants were instructed to assign a score from 0 (poor facial attractiveness) to 10 (excellent facial attractiveness). Informed consent was obtained from all the examiners prior to the experiment. 

## STATISTICAL ANALYSIS

Power analysis calculated at 92% yielded a minimum sample size of 68 participants per group. Data were retrieved and submitted to statistical analysis. 

ANOVA was applied to evaluate the mean scores given by the examiners according to the age, skin color, and schooling of the individuals in the images. Student’s t-test was used to compare gender differences. 

The responses of the participants about the most pleasant facial profile were analyzed with 95% confidence intervals (CI).

Student’s t-test was used to assess intra-examiner agreement for repeated scores and Cronbach’s alpha, to compare the statistical difference. Re-evaluations were carried out 15 days apart using 5% of the sample of examiners at random.

MiniTAB^®^ software (version 17) was used for all tests. A significance level of 5% (p<0.05) was set for all statistical analyses. 

## RESULTS

A total of 275 examiners answered the questionnaire. In intra-examiner agreement, 10 out of the 16 scores analyzed showed perfect agreement (100%). The lowest agreement was 97.5%. However, there was no statistical difference in the scores that varied between the first and second evaluations (p= 0.89). 

The examiners were classified according to category (oral and maxillofacial surgeons, orthodontists, and laypeople), age group, skin color, gender and education level. The results are listed in Table 1.

**Table 1:** Sample distribution according to category, age, skin color, gender and education.

SAMPLE	n	%
Category		
Oral and maxillofacial surgeons	91	24.93
Orthodontists	109	29.86
Laypeople	165	45.21
Age (years)		
18 - 35	137	37.53
36 - 55	198	54.25
Over 55	30	8.22
Skin color		
White	203	55.62
Brown	134	36.71
Black	22	6.03
Other	6	1.64
Gender		
Female	215	58.9
Male	150	41.1
Education level		
Secondary education	30	8.22
Undergraduate program completed	53	14.52
Graduate degree	282	77.26

In general, the most attractive face was that achieved by combined orthognathic surgery, followed by compensatory orthodontic treatment with mentoplasty and compensatory orthodontic treatment without surgery. The least attractive face was the untreated skeletal Class II. 

The relation between schooling and the aesthetic perception of the different simulated faces was evaluated. The mean scores, standard deviations, and significance levels for each simulation are shown in Table 2.

**Table 2:** Association between interviewees’ education level and facial attractiveness.

Education	Untreated Class II		Compensatory orthodontic treatment without surgery		Compensatory orthodontic treatment with mentoplasty		Combined orthognathic surgery	
	Median	SD	Median	SD	Median	SD	Median	SD
Secondary education	5.97^a^	3.23	6.50^a^	2.81	7.47^a^	2.42	8.97^a^	1.50
Undergraduate program completed	5.66^b^	2.40	6.45^b^	2.04	7.74^b^	1.57	7.94	1.84
Graduate degree	3.75^ab^	2.03	4.41^ab^	2.01	6.19^ab^	1.78	7.59^a^	1.88
P value*	0.000		0.000		0.000		0.000	

a / b: equal letters indicate statistically significant difference.

The results are different between participants holding graduate degree and the other groups. Statistical significance was observed in the graduate degree group, which gave lower scores to the videos than the groups with only undergraduate program completed or secondary education (p<0.05). The face generated by combined orthognathic surgery was the most attractive for all groups (p<0.05). 

Following the education criterion, the group of laypeople was evaluated separately to determine whether this factor also influenced the scores given within this group. In this group, graduate degree holders scored significantly lower than those who only undergraduate programs completed or secondary education (p<0.05).

**Figure 3:**
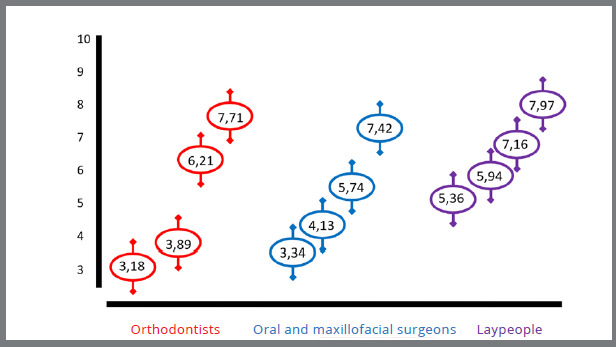
Comparison of interviewees regarding the most attractive face. ANOVA test. Significance level of p<0.05. * Statistically significant.

Finally, the association between the categories (laypeople, oral and maxillofacial surgeons, and orthodontists) and perceived attractiveness was assessed. Skeletal Class II face scores (compensatory orthodontic treatment and compensatory orthodontic treatment with mentoplasty) showed statistically significant differences between laypeople and the other two groups. Higher scores were given by laypeople (p<0.05) (Fig. 3).

Regardless of the category, the most attractive face was the one created by orthodontic treatment and combined orthognathic surgery, followed by compensatory orthodontic treatment with mentoplasty and compensatory orthodontic treatment without surgery. The untreated skeletal Class II was considered the least attractive. Laypeople scored higher than specialists, and the specialists scored similarly to each other. 

## DISCUSSION

The results revealed the negative aesthetic perception of the retrognathic profile, which is characteristic of skeletal Class II malocclusion. An improvement in facial attractiveness was observed when possible treatments were simulated, especially those involving skeletal correction obtained through orthognathic surgery. In addition, the perception of attractiveness was distinct between the groups of examiners. This indicates that professionals are more rigorous than laypeople in their assessments.

In this study, the face resulting from orthodontic treatment associated with combined orthognathic surgery (involving maxilla, mandible, and chin) was considered more aesthetically attractive. Likewise, Yüksel et al.^9^ and Pithon et al.^20^ observed that the profile generated by the increase in the chin-neck line as a result of skeletal Class II orthosurgical treatment was the most aesthetic. Unlike the convex profile, which was the least aesthetic. In contrast to our findings, Ghorbanyjavadpour and Rakhshan^7^ concluded that the convex profile was the preferred choice among the Iranian population. However, the results diverged due to cultural differences and the distinct facial features observed between the populations analyzed. 

Skeletal Class II is widely investigated due to its major functional and aesthetic alterations. Previous studies have assessed the outcomes of this malocclusion on the facial profile, as well as the attractiveness of corrections. However, none of them were as accurate in their simulations as this study, as we performed these corrections in real proportions using three-dimensional imaging scans. Although these studies evaluated similar aspects, the lateral photographs used are considered two-dimensional exams, thereby preventing the evaluation of these anteroposterior alterations from the frontal perspective.^9^ ^,^ ^19^ ^,^ ^20^

Profile silhouettes have been used in the literature to assess facial aesthetics. Similarly to photographs, this methodology hinders a dynamic facial assessment and increases the limitations of evaluating a static image even further, due to the simplicity of presentation to the examiner.^6^ ^,^ ^7^ ^,^ ^21^

Our team proposed an unprecedented resource for this type of assessment: the integrated use of advanced technologies such as CBCT scans, intraoral and facial scans. The use of the video format showing the head of the patient rotating from right to left allowed the examiners to dynamically analyze the face from different angles. This methodology was crucial in creating simulations that were as close as possible to real interpersonal contact. In fact, two-dimensional images, such as radiographs and photographs, are still widely used for correct and accurate diagnosis in orthodontics. However, three-dimensional digital resources are rapidly emerging due to the possibility of their integrated use, thus providing a diagnosis with greater detail and precision.^14^ ^,^ ^15^

The aesthetic perception of the population is a challenge that many researchers around the world have been trying to unravel for decades. The greatest limitation is undoubtedly the variability of the sample of evaluators, which can be influenced by schooling, for example.

Considering that individuals with more schooling have broader access to information and therefore a greater capacity for decision-making, the question arises if these aspects could influence how these people perceive changes in facial aesthetics. Thus, this study assessed the responses of individuals with different education backgrounds: high school, undergraduate, and graduate. The results indicated that the greater the level of schooling, the more rigorous the assessments, as demonstrated by the lower grades scored. Similarly, Hönn et al.^22^ reported that individuals who had completed higher education were more critical in their evaluations than those with a lower level of education.

Attractiveness studies should therefore not only investigate the perception of laypeople, but also of the professionals involved in the aesthetics alterations investigated. Understanding whether the preference of these audiences is compatible with their education is an essential factor in the aesthetic success of the proposed treatments. Studies point to a lack of consensus between dentists from different specialties and between dentists and laypeople. This indicates the need for more in-depth studies on the subject to better understand the aesthetic standards of the population.^21^ ^,^ ^23^

The sample for this study comprised orthodontists, oral and maxillofacial surgeons and laypeople in dentistry. Orthodontists are more sensitive at detecting changes,^11^ while oral and maxillofacial surgeons deal directly with altered dentoskeletal positioning. In many clinical situations, these two groups of specialists need to collaborate to provide treatments with satisfactory results. The third group is composed of patients who could possibly receive these treatments: the laypeople, who represent the universe of individuals without specific technical knowledge. These individuals are of considerable importance, as their opinion will guide changes in the evaluation standards that are currently in force.^24^

The perception of professionals was similar and more rigorous, as they scored lower than laypeople. This finding was already expected, as professionals are likely to have a more accurate perception of facial changes due to their technical knowledge. Previous studies have also found a more rigorous evaluation by professionals, compared to laypeople.^25^ ^,^ ^26^

Regardless of the category, the most attractive face was the one created by orthodontic treatment and combined orthognathic surgery, followed by compensatory orthodontic treatment with mentoplasty and compensatory orthodontic treatment without surgery. The untreated skeletal Class II was considered the least attractive. Despite the difference in technical knowledge, laypeople prefer the result that generates the most anteroposterior changes, as well as a straighter profile. This finding highlights the need for orthodontic-surgical treatment not only for functional but also for aesthetic purposes. 

Correspondingly, Yuksel et al.^9^, in a sample of laypeople evaluators, reported that the most accepted profile was that achieved by changes following orthodontic treatment with orthognathic surgery. Unlike our results, Tsiouli et al.^24^ found no differences when comparing the assessment of professionals and laypeople. We emphasize that these authors evaluated facial alterations resulting from the use of orthopedic appliances, which generate less significant changes when compared to those produced by orthognathic surgery.

To our knowledge, this is the first study to combine three-dimensional scans, CBCT, facial scans and digital dental models from the same patient to generate dynamic 3D simulations for attractiveness assessment. It’s interesting to enable treatment simulations with high accuracy of movements to assess skeletal Class II attractiveness perception and possible corrective treatments. However, some limitations may be associated with the methodology, such as the quality of the face scan images, the non-inclusion of images of the female gender, as well as different skin colors. 

The use of 3D images is a reality in clinical practice for diagnostic purposes, but it can go much further with the possibility of discussing possible results with much more predictability, especially with the advancement of technologies. The possibility of using simulations of results with greater precision is undoubtedly considered a great advance for greater assertiveness of the proposed treatment, not only in functional quality, but also in aesthetic gain. 

Therefore, more studies using this methodology are needed to improve the results and increase understanding of facial aesthetics. Ultimately, this will have a positive impact on professional planning and provide patients with a better quality of life.

## CONCLUSIONS

The results of this study indicated that:

In the three-dimensional analysis of facial aesthetics, all the examiners considered untreated skeletal Class II to be the worst result. Laypeople were less rigorous in their assessments than orthodontists and oral and maxillofacial surgeons, who scored similarly. Another important finding was the Individuals with higher levels of education are more rigorous in their aesthetic evaluation, giving lower scores. For all the examiners, the most attractive face was created by orthodontic treatment and combined orthognathic surgery, followed by compensatory orthodontic treatment with mentoplasty and compensatory orthodontic treatment without surgery. This outcome shows evidence of the positive impact of the treatments on facial aesthetics. Therefore the use of 3D images to evaluate the aesthetic perception of facial changes and possible treatments presents itself as an alternative closer to reality, thus promoting the possibility of even more satisfactory clinical results.
